# Assessing synergistic effect of Jerusalem Artichoke juice and antioxidant compounds on enhanced viability and persistence of *Bifidobacterium* species, palatability, and shelf life

**DOI:** 10.1002/fsn3.2815

**Published:** 2022-03-21

**Authors:** Mustafa A. M. Alsharafani, Taghreed Abdullah, Zahraa A. Jabur, Abdulwahed Ahmed Hassan, Abeer S. Alhendi, Amir Abdulmawjood, Izhar U. H. Khan

**Affiliations:** ^1^ Ministry of Science and Technology, Directorate of Environment and Water Research Food Pollution Center Baghdad Iraq; ^2^ Department of Veterinary Public Health (DVPH) College of Veterinary Medicine University Mosul Mosul Iraq; ^3^ Agriculture and Agri‐Food Canada Ottawa Research and Development Centre Ottawa Ontario Canada; ^4^ Department of Quality Control Grain Board of Iraq Altaji, Baghdad Iraq; ^5^ Institute of Food Quality and Food Safety University of Veterinary Medicine Hannover Hannover Germany

**Keywords:** antioxidants, *Bifidobacterium* spp., inulin, Jerusalem Artichoke juice, shelf life, viability

## Abstract

Commercial vegetable and fruit juices with probiotics are new functional type of beverages; however, limitations including persistence and impact of probiotic bacteria on palatability and shelf life may prevent their industrial development. This study evaluated the effect of antioxidant compounds (ascorbic acid, astaxanthin, and ginseng) on viability and persistence of *Bifidobacterium* spp. in Jerusalem Artichoke (JA) juice; and determine the impact of these antioxidants on the sensory (color, texture, flavor, acidity) properties, free reducing sugar (inulin and fructose), and shelf life in the fortified JA juice. Overall, the JA juice fortified with ascorbic acid showed a significant impact on the rate of persistence of two targeted bifidobacterial strains from 1 to 28 days at 5°C. Both strains produced slight acidity in ascorbic acid fortified JA juice as compared to other tested samples. Similarly, the JA juice fortified with ascorbic acid showed a significantly high increase in the total number of bifidobacterial cells of both species, enhanced palatability, and shelf life as compared to astaxanthin and ginseng extract. The quadratic model indicated a strong association between ascorbic acid, ginseng extract, and astaxanthin with a bifidobacterial cell concentration in the fortified JA juices. The Box–Behnken design was considered a feasible analysis for describing fortified JA juice and the rate of viability and persistence of bifidobacteria during 28 days of storage at 5°C in all trials. In conclusion, JA juice fortified with ascorbic acid showed a significant impact on improving the cell viability and persistence of probiotic bacteria, enhanced palatability, and shelf life as compared to other compounds tested.

## Graphical abstract

Functional food has now been emerged as a vital part of food industry since these food products not only offer nutritional functions and health advantages, but also help in mitigating risk of chronic illness. The study has provided information on synergistic effect of Jerusalem Artichoke juice with antioxidant compound (ascorbic acid) significantly improve the viability and persistence (˃107 CFU/ml) of probiotic bacteria (*Bifidobacterium* species) and shelf life (up to 28 days at 5°C). The data analysis for scaling up the optimal circumstances from laboratory to a large‐scale can be applied in the food industry.

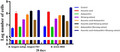

## 1 INTRODUCTION

Jerusalem Artichoke (JA) (*Helianthus tuberosus*) also called sunroot or sunchoke, originated from North America, has been classified as a vegetable. The plant is an erect, rhizomatous perennial herb that can be distinguished by its sunflower‐like (*Helianthus annuus*) appearance (Acevedo‐Martínez et al., 2018). Currently, the JA is cultivated in Australia, New Zealand, and many North American, Northern European, Asian countries (Kays & Nottingham, 2008). The JA tubers (roots), taste like Artichoke, are the edible part of the plant and consumed in raw or cooked form similarly as potatoes, stewed, soup, puree, or salad. In Iraq, the tubers are traditionally used as pickled or emergency food as well as a substitute for coffee. However, nowadays, these are used as a trendy vegetable (Bach et al., 2013).

The JA tubers contain ~78% moisture, 12.8% carbohydrate (mainly inulin 9.6% and fructose 3.2%), 2%–6.1% protein, 0.5% minerals, 1.98% fiber, and 0.01% low total lipids (Alsharafani, 2006; USDA, 2018). A 100 g of tubers is rich source of potassium (429 mg), phosphorus (78 mg), magnesium (17 mg), calcium (14 mg), sodium (4 mg), iron (3.4 mg), copper (0.14 mg), zinc (0.12 mg), manganese (0.6 mg), and selenium (0.7 µg). Similarly, 100 gm of tubers contains various vitamins, including ascorbic acid (4 mg), thiamin (0.2 mg), riboflavin (0.06 mg), niacin (1.3 mg), pantothenic acid (0.397 mg), and B6 (0.077 mg) (Celik et al., 2013; USDA, 2018). Among carbohydrates, inulin (polyfructans), a polysaccharide that has many health benefits where the unique microorganisms (*Lactobacillus* and *Bifidobacterium*) efficiently break it down in the large intestine which helps to boost optimal intestinal quality and reduces intestinal inflammation (Apolinário et al., 2014; Blauer, 1989). Considering its health benefits, a mutualistic cocktail was made from raw JA juice (Apolinário et al., 2014). JA enhances probiotics and dietary fiber effect that facilitates the growth of beneficial bacteria and improve digestive health. Also, it may serve as sugar or fat replacement in food and promotes mineral absorption (Munim et al., 2017). Inulin can directly influence the hormones that regulate appetite. Inulin enhances glucose consumption and raises glycogen synthesis with the key factor in preserving natural glucose content. It also has the function of helping liver to remove contaminants (Alsharafani et al., 2007; Roberfroid, 2007).

Commercial juices with probiotics have, recently, gained popularity among health and wellness circles; however, certain limitations such as persistence of probiotics and shelf life may prevent their industrial development. In recent years, consumers have raised their consumption of nondairy probiotic products such as fermented soy products and vegetable and fruit juices (Acevedo‐Martínez et al., 2018). The consumption of these type of products have been substantially increased since they are a high source of enzymes, vitamins, fibers, and minerals as well as the bioactive compounds may enhance their absorption as compared to the raw or fresh plant tissues (Havas et al., 2014; Towviriyakul et al., 2012). Therefore, maintaining the viability of probiotic bacteria is an essential requirement to mitigate adverse effects in these fresh food products (Nualkaekul et al., 2011; Perricone et al., 2015). Adding *Bifidobacterium* spp. to food is restricted due to their susceptibility to aerobic and acidic conditions. Moreover, low pH and aerobic environment inhibit viability and persistence of bifidobacteria, particularly in vegetable and fruit matrices. The incorporation of ascorbic acid in food can, however, reduce the amount of oxygen stress for the *Bifidobacterium* activities (Talwalkar & Kailasapathy, 2004). Similarly, ginseng (*Panax ginseng* root c.a. Mayer) contains many bioactive compounds (e.g., ginsenosides). The ginsenoside sugar chains are strongly connected to their functionality. The pharmacologically fermented ginseng with bifidobacteria and lactic acid bacteria has been identified in the biotransformation of ginsenosides (Hong et al., 2011; Lu et al., 2009). Moreover, astaxanthin has a specific molecular structure characterized by the presence of oxygen as a hydroxyl group that creates a powerful antioxidant (Bolin et al., 2010). Considering the benefits of ascorbic acid, ginseng, and astaxanthin, JA juice fortified with these antioxidant compounds and probiotic bacteria with no added sugars would be a delicious healthy option over low‐calorie (e.g., orange) juices (Serpen, 2012). Similarly, fermented milk beverages made from JA inulin could be a better alternative drink.

Considering the health benefits of JA, the present study was designed to evaluate the effect of three antioxidant compounds, including ascorbic acid, astaxanthin, and ginseng extract on the viability and persistence of *Bifidobacterium breve* and *B. longum* subsp. *longum* in JA juice under aerobic and low pH conditions to maintain and improve sensory properties and shelf life at 5ºC. Secondly, assessing the impact of these antioxidants on acidity, inulin, and fructose in the fortified JA juice. Box–Behnken experimental design for response surface methods applied to explain the study design which scales up the innovative JA juice in the food industry that cannot be applied in conventional analyses at laboratory‐scale conditions.

## 2 MATERIAL AND METHODS

### 2.1 Preparation of JA juice

Juice from fresh Jerusalem Artichoke (*H. tuberosus*) tubers, obtained from the local market, was extracted using the following procedure: 2 kg of tubers was washed, cleaned, and boiled in 1‐L water at 85°C for 15 min for blanching of the tuber fruit. The cooked tubers were sliced into small pieces (1–2 cm^3^) and mashed in a blender 600w bl‐60phnmy (Toshiba). The smoothie was filtered and pressed according to the procedure described by Kays and Nottingham (2008) for removing dregs. The total soluble solids were 15° Brix after the juice was diluted to a 1:1 ratio (v/v) with sterile water. The final juice was sterilized at 85°C for 15 min, cooled at 25°C, and stored at 5°C for further analysis (Bampidis et al., 2019; Lee et al., 2018).

### 2.2 Ginseng extract preparation

Ginseng extract was prepared from commercial Korean ginseng powder (*P. ginseng* C. A. Meyer) (NaturaHouse) as described by Bolin et al. (2010). Ethanol 70% was used as an extraction solvent for heat reflux extraction. The ginseng extract (GE) was collected after 6 h during the heat reflux process when the temperature reached at 80°C. The GE was separated from the extraction solvent with a rotary evaporator vacuum (RV‐3, IKA^®^‐Werke) and dried at 45°C in the vacuum dryer (Kambic‐Vakuumtrockner‐CiK Solutions). The dried GE was milled and the powder was kept at −18°C until used for the assay.

### 2.3 Fortified JA juice preparations

According to daily required intake (Bampidis et al., 2019; Lee et al., 2018), ascorbic acid, astaxanthin, and ginseng extract were prepared at various concentrations (15, 12, and 100 mg/L). Seven combinations of JA juice with individual or multiple antioxidants including JA juice only with ascorbic acid, astaxanthin, or ginseng extract, JA juice with ascorbic acid and astaxanthin extracts, JA juice with ascorbic acid and ginseng extract, JA juice with astaxanthin and ginseng extract, and JA juice with ascorbic acid, astaxanthin, and ginseng extract. In addition, JA juice without an antioxidant compound was used as a control.

### 2.4 Total carbohydrate analysis

Total carbohydrates as free reducing sugar (fructose) and inulin, in the JA juice, were determined using a colorimetric method. Major carbohydrates (Inulin and fructose) in the JA juice were determined using the free reducing sugars method with 3.5‐dinitrosalicylic acid (DNS) as described previously (AOAC, 1980; Chaplin & Kennedy, 1994). The optical absorption value was measured at 540 nm by SPUV‐19‐Spectrophotometer (SCO‐Tech). The standard curve of fructose was determined using the following formula:

(1) γ=0.304x+0.0324

where γ, absorbance; x, concentration; *R* ^2^ = .9924: coefficient of determination.

To determine the inulin concentration (g/L), the juice was hydrolyzed to release inulin in the form of fructose (Alsharafani, 2006). Briefly, hydrochloric acid solution (5%) was added to 100 ml juice to obtain 1% final concentration followed by boiling for 50 min. The hydrolyzed solution was neutralized with NaOH (1N), to obtain reduced sugar formed as fructose equivalent, which contains carbohydrate expressed as inulin. Inulin concentration (g/L) was estimated by calculating the difference of fructose concentration before and after hydrolysis as described previously (AOAC, 1980; Chaplin & Kennedy, 1994) using the following equation:

(2) Inulin=f×1.2

where *f*: fructose equivalently formed; 1.2: conversion factor.

### 2.5 Sensory criteria and assessment

The sensory properties of the fortified JA juice after adding bifidobacterial strains were assessed as previously described by Poste (1991). Based on preliminary satisfactory sensory acceptance from nine experienced panelists, the sensory evaluation criteria including taste, consistency, color, and smell of the juice mixtures, and the tested juices were graded as: 4.1–5.0 very good; 3.1–4.0: good; 2.1–3.0: satisfactory; 1.1–2.0: sufficient; and 0.0–1.0: not acceptable.

The sensory evaluation process was carried out by expert panelists from the Department of Food Sciences, University of Baghdad. The panelists followed standardized sensory criteria, measurements and made objective decisions without any personal preferences. Each panelist detected, recognized, and agreed upon common procedure and exact connotation of each descriptive term by using evaluation techniques for odor, appearance, flavor, and texture criteria. The samples were provided in plastic cups at 20°C. The taste, color, quality, consistency, and general acceptability of the food were taken into consideration while making the final decision.

### 2.6 Preparation of fortified juices with bifidobacterial strains

The *B. breve* M4A and *B. longum* subsp. *longum* FA1 reference strains with functional properties previously reported (Alsharafani et al., 2016) were subcultured in MRS medium broth (Merck KGaA) supplemented with l‐cysteine (0.5 g/L) (Merck KGaA) incubated at 37°C under anaerobic conditions for 24 h using anaerobe Jar and AnaeroGen GasPak System (Thermo Scientific™ Oxoid). The broth was centrifuged at 2500 *g* for 15 min at 4°C (Allegra 64R, Refrigerated). The pellet was washed twice with phosphate‐buffered saline (PBS) (0.1 mol/L pH 7.4) and resuspended in PBS. The cell suspensions 10% (v/v), form cell density (~10^9^ CFU/ml), of both bifidobacterial species were prepared and mixed with six different combinations of JA juice mixtures, incubated at 37°C for 12 h, and transferred to 5°C for 4 weeks (Alsharafni et al., 2015, 2016). The viable cell concentration of each bifidobacterial species was measured from day one to last day of storage on MRS agar supplemented with l‐cysteine (0.5 g/L) by incubating at 37°C under anaerobic condition for 72 h using 10‐fold serial dilution assay. The bifidobacterial cells were further confirmed and counted based on colony morphology and microscopic analysis after incubation of Nutrient agar plates at 5, 27, and 37°C under aerobic and anerobic conditions, respectively.

### 2.7 Experimental design

Two levels and three‐factor full factorial experiments were established using the Box–Behnken design (BBD). Based on 14 factorial points (Center point run) and three replicates (bacterial cell concentration) at the central point, full factorial BBD was carried out for three independent variables (ascorbic acid, astaxanthin, and ginseng extract) (Anderson & Whitcomb, 2017). The response surface method (RSM) was applied to assess the optimum juice supplementation for improved viability and persistence of bacterial cells in the fortified JA juices (Anderson et al., 2015, 2018). The independent and multiple variables were symbolized as: ascorbic acid: *X*_1_; astaxanthin: *X*_2_; ginseng extract: *X*_3_; ascorbic acid and astaxanthin: *X*_1_ *X*_2_; ascorbic acid and ginseng extract: *X*_1_ *X*_3_; astaxanthin and ginseng extract: *X*_2_ *X*_3_; and ascorbic acid, astaxanthin, and ginseng extract: *X*_1_ *X*_2_ *X*_3_. Cell concentration of *B. breve* M4A or *B. longum* subsp. *longum* FA1 strain was used as a dependent variable. The RSM was fitted to each of the response (or dependent *Y*_1_ and *Y*_2_) variables according to Design Expert (version 12; Stat‐Ease Inc.) using the following equation:

(3) $$\begin{array}{cc} Y & =\beta_{0}+\beta_{1}X_{1}+\beta_{2}X_{2}+\beta_{3}X_{3}+\beta_{12}X_{1}X_{2}\\ & +\beta_{13}X_{1}X_{3}+\beta_{23}X_{2}X_{3}+\beta_{11}X_{1}^{2}+\beta_{22}X_{2}^{2}\\ & +\beta_{33}X_{3}^{2}+\beta_{13}X_{1}^{2}X_{3}+\beta_{12}X_{1}X_{2}^{2}+\varepsilon \end{array}$$

where *Y*: Dependent response variable (expressed as log_10_); *β*_0_, *β*_1_, *β*_13_, and *β*_23_: Estimated regression coefficients, where *β*_0_ is an intercept; *β*_1_, *β*_2_, and *β*_3_: Linear effects; *β*_11_, *β*_22_, and *β*_33_: Quadratic effects; *β*_12_, *β*_13_, and *β*_23_: Interaction effects and the residue (*ε*).

For developing prediction models for each dependent variable, regression analysis was applied to calculate the coefficient of determination (*R* ^2^). Overall statistical data analysis was performed using Design Expert (version 12; Stat‐Ease Inc.). Generally, a BBD was employed using Design‐Expert software according to the selected variables. The practical range and level of the identified factors were outlined. The statistical analysis was performed based on the concentration of the predicted factors coded as −1: low; 0: medium; and +1: high levels. According to a second‐order BBD, 17 experiments including 14 factorial and three center point runs as replicates for experimental error estimation with three independent variables were performed. The experimental design in the coded and actual levels is described in Table 1.

**TABLE 1:** Box–Behnken design (BBD) data of fortified JA juices for three variables of the independent factors with experimental values of each *Bifidobacterium breve* M4A (*Y*_1_) and *Bifidobacterium longum* subsp. *longum* FA1 (*Y*_2_) strain for, a dependent factor, response during 4‐week storage at 5°C

Run	Factor 1 = *X*_1_ (10 mg/L)	Factor 2 = *X*_2_ (12 mg/L)	Factor 3 = *X*_3_ (100 mg/L)	Response *Y*_1_ cell log no. (CFU/ml)	Response *Y*_2_ cell log no. (CFU/ml)
1	0	0	0	8.318	8.017
2	1	1	0	8.428	8.157
3	1	−1	0	7.791	8.167
4	−1	0	−1	6.862	6.826
5	0	1	−1	7.631	7.733
6	1	0	−1	6.842	7.821
7	0	−1	1	7.623	7.977
8	0	0	0	8.268	8.069
9	0	1	1	8.257	8.007
10	0	0	0	8.318	8.021
11	−1	1	0	7.245	7.345
12	−1	−1	0	7.417	7.341
13	−1	0	1	7.311	7.411
14	0	0	0	8.268	8.021
15	1	0	1	8.518	8.249
16	0	0	0	8.247	8.047
17	0	−1	−1	7.953	7.676

### 2.8 Fitness model and statistical analyses

The evaluation of RSM model was performed by using statistical analysis of BBD. Three main tests, including the significance of terms, regression model, and lack‐of‐fit, were applied to assess the fitness and reliability of a model (Anderson & Whitcomb, 2017). A lack‐of‐fit test augmented to screen described the model design to a response surface design to better model the relationship between the factors and the response. Based on the experimental data for obtaining the regression equation, multiple polynomial fitness models including linear, two factorial, quadratic, and cubic models were applied using ANOVA test. Based on the significance of terms, *f*‐ and *p*‐values were used to determine the most effective factor in the response of interest. Higher *f*‐value along with lower *p*‐value (normally ˂0.05) of the model terms were considered as a crucial effect on the response. The lack‐of‐fit test was used to identify an unsuccessful model where the data were excluded from the regression equation and assess the fitness and reliability of the model. The significant model suggests that the residual error did not exceed pure error, and consequently the model was significant. To verify the adequacy of the model, determination coefficient *R* ^2^, adjusted *R* ^2^, and predicted *R* ^2^ were applied. The *R* ^2^ value represents the proportion of total variations in experimentation, whereas adjusted *R* ^2^ was the variation of mean presented by the model in which a number of variables of data set was taken into account. Typically, a good model should have an *R* ^2^ value close to one, and this indicates that better fitness between existing data and the empirical model is achieved. Additionally, power calculations were performed using response type “Continuous” at a 5% *α*‐level to detect the specified signal/noise ratio of the difference to detect Delta (*δ* signal) and estimated standard deviation Sigma (noise). A factorial effect alias was not found for the performed model. The reproducibility of the model was tested using the coefficient of variation (CV). The effect of individual variables and their interaction effect on the response were additionally investigated using three‐dimensional response surface plots. Finally, the proposed model was used to define and verify the appropriate conditions of each supplement to obtain the best viable cell concentration.

The carbohydrate and pH data obtained from triplicate runs and duplicate analysis were statistically analyzed using ordinary one‐way analysis of ANOVA as well as Tukey's multiple comparisons tests. The mean and standard deviation (SD) data are presented in this study. The GraphPad Prism program (v8.0 software, USA) was used for data processing, where *p* < .05 was considered as statistically significant.

## 3 RESULTS AND DISCUSSION

### 3.1 Fortified Jerusalem Artichoke: a promising juice

The natural amount of sugar per serving of the Jerusalem Artichoke (JA) juice is slightly less than the natural sugar in grape and orange juice (Serpen, 2012; Towviriyakul et al., 2012). Most dietary fiber in JA juice comes from inulin and fructooligosaccharides which act as probiotics and are considered as an alternative diet for people who are at risk for less dietary fiber consumption, especially the elderly community (Towviriyakul et al., 2012). In the present study, the JA juice was prepared as a novel beverage from the tubers of the plant. The new JA juice fortified with ascorbic acid successfully improved bacterial viability maintained bacterial cell concentration of *B. breve* strain M4A and *B. longum* subsps. *longum* strain FA1 and enhanced palatability. Preheating treatment of the JA tubers inhibited the browning reaction enzymes that hindered the development of color pigments during the process of extracting JA juice. The preheating treatment of JA tubers also helps in maintaining the color during JA juice production (Bach et al., 2013; Jeong et al., 2011; Kim et al., 2010). Additionally, the JA tubers were subjected to the preheating treatment to obtain a healthy and flavorful drink, preserving distinctive freshness as well as better sensory properties. This also substantially impacts the shelf life of juice (Cassano et al., 2007). Most vitamins degrade while heating that leads to decrease effectiveness. The heat often helps to prevent off‐flavors (Braddock & Goodrich, 2002). The JA freshness is due to volatile organic compounds known as ethyl acetate, ethyl butyrate, and terpene thiols where terpene is the most common compound, and β‐bisabolene is one of the dominant compounds of a terpene. In addition, hexanal is the highest and aldehydes is the second most abundant compound (Jung & Shin, 2017).

### 3.2 Carbohydrate fermentation analysis

Fiber has numerous health benefits where weight regulation is the most common since inulin enhances glucose consumption and raises glycogen synthesis; therefore, inulin can directly influence the hormones and regulate appetite. However, a slow‐burning carbohydrate can help in the ability to regulate appetite without the need for complex and often difficult diet adjustment. Overall, fructose can act as a great source of energy where inulin aids in the production of protein, bile acids, and cholesterol as well as it has function to help in removal of contaminants from liver with regard to health benefit that may impact on improving human health (Alsharafani et al., 2007; Alsharafani & Al‐Nouri, 2009; Roberfroid, 2007).

**FIGURE 1:**
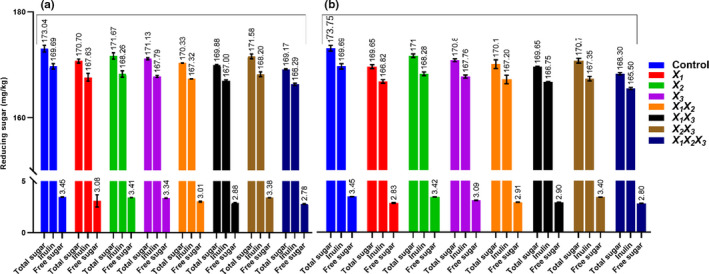
Data showing total sugar, free sugar (fructose), and inulin contents in individual and combination of fortified JA juice samples after 12 h of fermentation at 37°C and stored at 5°C for 4 weeks

To determine the content of free fructose and fructose produced by hydrolysis of inulin, the reaction between fructose and DNS reagent was estimated. Total sugar in the JA juice simulated with *B. breve* M4A was 173,14 ± 0.52, 170.70 ± 0.38, 171.66 ± 0.61, 171.13 ± 0.19, 170.33 ± 0.05, 169.88 ± 0.81, 171.58 ± 0.45, and 169.07 ± 9.24 mg/kg for control, *X*_1_, *X*_2_, *X*_3_, *X*_1_ *X*_2_, *X*_1_ *X*_3_, *X*_2_ *X*_3_, and *X*_1_ *X*_2_ *X*_3_ samples, respectively. A combination of fortified JA juice *X*_1_ *X*_2_ *X*_3_ contained a significantly (*p* < .05) low rate of free fructose (2.78 ± 0.04 mg/kg) as compared to the control (3.45 ± 0.02 mg/kg) sample containing *B. breve* M4A strain (Figure 1a). In contrast, free fructose concentration at the rate of 3.07 ± 0.58, 3.41 ± 0.01, 3.34 ± 0.02, 3.01 ± 0.04, 2.88 ± 0.03, and 3.38 ± 0.01 mg/kg was recorded in various combinations (*X*_1_; *X*_2_; *X*_3_; *X*_1_ *X*_2_; *X*_1_ *X*_3_; and *X*_2_ *X*_3_) of fortified JA juice samples, where no significant difference was observed between tested and control samples. The inulin concentration (169.69 ± 0.50 mg/kg) in control JA juice was significantly (*p* < .05) higher than the single and various combinations (*X*_1_: 167.62 ± 0.74; *X*_2_: 168.25 ± 0.60; *X*_3_: 167.79 ± 0.20; *X*_1_ *X*_2_: 167.32 ± 0.02; *X*_1_ *X*_3_:167.00 ± 0.15; *X*_2_ *X*_3_: 168.20 ± 0.45; and *X*_1_ *X*_2_ *X*_3_: 166.29 ± 0.23 mg/kg) of fortified JA juice samples. Among the fortified JA sample, the inulin concentration of the *X*_1_ sample was significantly (*p* < .05) lower than other fortified JA juice samples. On the other hand, *X*_1_ *X*_2_ *X*_3_ fortified JA juice sample had a significantly (*p* < .05) higher concentration than other samples.

Whereas total sugar in the JA juice simulated with *B. longum* subsp. *longum* FA1 was 173.14 ± 0.52, 169.65 ± 0.37, 171.70 ± 0.34, 170.84 ± 0.27, 170.10 ± 0.82, 169.64 ± 0.06, 170.75 ± 0.44, and 168.30 ± 0.22 mg/kg for control, *X*_1_, *X*_2_, *X*_3_, *X*_1_ *X*_2_, *X*_1_ *X*_3_, *X*_2_ *X*_3_, and *X*_1_ *X*_2_ *X*_3_ samples, respectively. The fermented carbohydrates of fortified JA juice samples *X*_3_, *X*_1_ *X*_2_, and *X*_1_ *X*_3_ containing *B. longum* subsp. *longum* FA1 strain had low free fructose (3.08 ± 0.02, 2.90 ± 0.02, and 2.89 ± 0.02 mg/kg) and inulin (167.75 ± 0.30, 167.20 ± 0.80, and 166.750 ± 05 mg/kg, respectively) showed significantly (*p* < .05) high fermentation as compared to *X*_2_, *X*_2_ *X*_3_, and control samples in fructose (3.42 ± 0.2, 3.40 ± 0.02, and 3.45 ± 0.03 mg/kg) and inulin (168.28 ± 0.34, 167.35 ± 0.42, and 169.69 ± 0.50 mg/kg) concentration, while significantly (*p* < .05) high carbohydrate fermentation of fructose (2.82 ± 0.04 and 2.79 ± 0.02 mg/kg) and inulin (166.83 ± 0.33 and 165.50 ± 0.21 mg/kg) was recorded in *X*_1_ and *X*_1_ *X*_2_ *X*_3_ than other tested samples (Figure 1b).

The capacity of carbohydrate fermentation in the juice has previously been demonstrated by *Bifidobacterium* species where they show an ability to grow on simple and complex carbohydrate fluid media such as juices (Milani et al., 2015; Selak et al., 2016). Previously, Milani et al. (2015) reported that *Bifidobacterium* strains induce a high rate of fermentation in sour cherry and orange juice. Therefore, in the present study, fortified JA juice samples either with single or multiple antioxidant compounds (*X*_1_, *X*_2_, or *X*_3_) showed a high rate of carbohydrate assimilation during fermentation than the control sample. Our results are in congruence with a previous study where a significant increase in cell concentration (ranging from 6.2 × 10^6^ to 1.8 × 10^9^ CFU/ml) of *B. longum* strain Bb‐46 in orange, carrot, and tomato juices was observed after 24 h of fermentation (Havas et al., 2014). In contrary, the growth rate of *B. bifidum* NCFB 1454 in sour cherry juice and orange juice was not significant in contrast to tomato juice samples fermented with *B. longum* Bb‐46, *B. bifidum* B3.2, and *B. longum* A4.8 strains (Goderska et al., 2007). Our study results are supported by other studies where pre‐ and probiotic bacterial (*Lactobacillus acidophilus* and *Bifidobacterium animalis* subsp. *Lactis*) species were used for fermentation in food products that could be applied to the industrial scale (Shafi et al., 2019; Zacarias et al., 2020).

### 3.3 Acidic production

**FIGURE 2:**
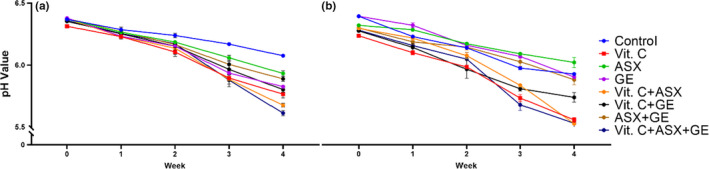
pH values of JA juice and supplemented juice incubated at 37°C for 18 h and 4‐week storage at 5°C; (a) *Bifidobacterium breve* M4A, (b) *Bifidobacterium longum* subsp. *longum* FA1

Initially, the pH value of the control JA juice sample simulated with *B. breve* M4A and *B. longum* subsp. *longum* FA1 strains was 6.4 which decreased to 5.98 and 6.07, respectively. After 28 days of incubation at 5ºC, the fortified JA juice samples fermented with *B. breve* M4A strain had pH 6.34 decreased to pH 5.90, 5.87, 5.82, 5.78, 5.52, and 5.5 up to 5.44 for *X*_2_, *X*_2_ *X*_3_, *X*_3_, *X*_1_, *X*_1_ *X*_2_, *X*_1_ *X*_3_, and *X*_1_ *X*_2_ *X*_3_, respectively (Figure 2). Likewise, the initial pH value of fortified JA juice samples spiked with *B. longum* subsp. *longum* FA1 strain was 6.35 which is decreased to pH 5.94, 5.86, 5.74, 5.52, 5.5, and 5.45 up to 5.44 for fortified JA samples *X*_2_, *X*_2_ *X*_3_, *X*_3_, *X*_1_ *X*_2_, *X*_1_ *X*_3_, and *X*_1_ *X*_2_ *X*_3_, respectively. During the storage period, changes in the pH values of JA juice were detected (Figure 2). This indicates that fermented JA juice might have low buffering capacity. However, a minimum fermentation of the *X*_2_ fortified JA juice sample was observed as compared to the other tested samples. In contrast, carrot juice might be deficient in other nutrients essential for *Bifidobacterium* growth. The inclusion of a fermentable source of carbohydrates or enzymes that make fermentable carbohydrates or other growth factors for enhancing the growth of bacteria. A previous study reported that carrot juice has shown a good medium for the growth of *Bifidobacterium lactis* and *Bifidobacterium bifidum* strains as well as *Lactobacillus rhamnosus* and *Lactobacillus bulgaricus* (Kun et al., 2008); however, other study findings have shown that the fermented carrot juice is a suitable medium for probiotic lactobacilli but not bifidobacteria although it may change lactobacilli phenotypic characteristics (Tamminen et al., 2013).

These selected products were abundant in bioactive (flavonoid and carotenoid) compounds. This enabled us to investigate the essence of shifts of antioxidant function during a fermentation process. In the presence of bifidobacteria, in most instances, antioxidants potentially decrease during the fermentation process. Previous study showed that the amount of antioxidants decreased only in the JA juice simulated with the *B. lactis* B b‐12 strain. This finding is promising since the compounds may retain their functionality in the fermented juice and can be used to design a novel functional food product (Havas et al., 2014). Fermentation of juices (Jerusalem Artichoke, pineapple, pumpkin, spinach, and cucumber) simulated with *L. acidophilus* DSM13241, *L. paracasei* subsp *paracasei* ATCC 55544, *L. rhamnosus*, *L. plantarum*, and *B. animalis* subsp. *lactis* BB‐12 at 37°C for 24 h showed an increase in viable probiotic cells up to 9.42 log CFU/ml (Güney & Güngörmüşler, 2021).

Moreover, JA juice fortified with ascorbic acid (*X*_1_) simulated with *B. breve* M4A or *B. longum* subsps. *longum* FA1 species produced high acidity compared to ginseng extract (*X*_3_), while astaxanthin (*X*_2_) produced a low rate of acidity, which could be due to low solubility in water and considered as a limiting factor (Gross & Lockwood, 2004). These facts indicate that the persistence of the *Bifidobacterium* strains is dependent on various factors, including synergistic effect with carbohydrates and antagonistic with the acidity and oxygen. Also, these results were in agreement with a previous study on carrot juice fermentation fortified with inulin and *Bifidobacterium* BB12, and *L. acidophilus* LA‐5 (Buruleanu et al., 2009) with an increase in reduction of sugar intake ratio and decrease in pH during 48 h of fermentation (Buruleanu et al., 2009). Moreover, Acevedo‐Martínez et al. (2018) reported the acidity of the mango juice with *Lactobacillus casei* and 5% fructooligosaccharides where a significant change in acidic pH after 1 week at 4°C was observed.

### 3.4 Application of regression model

The statistical analyses were used to perform the experimental data regression analysis and to draw the surface plot of the response. The statistical parameters illustrated in Table 2 were estimated using the ANOVA test for *B. breve* M4A and *B. longum* subsp. *longum* FA1 strains, respectively. The quadratic model was not aliased and the evaluation was ensured the adequate design which can tidily estimate the interactions of interest as the performance presented by the Design‐Expert program. The final empirical model in terms of a coded factor for the colony‐forming unit (CFU/ml) shown in Equations (2) and (3) for *B. breve* M4A and *B. longum* subsp. *longum* FA1 is given below:

(4) $$\begin{array}{cc} Y_{1} & =8.28380+0.296750X_{1}+0.097125X_{2}+0.074000X_{3}\\ & +0.202250X_{1}\times X_{2}+0.306750X_{1}\times X_{3}+0.239000X_{2}\times X_{3}\\ & +0.523150X_{1}^{2}‐0.040400X_{2}^{2}‐0.377400X_{3}^{2}\\ & +0.457250X_{1}^{2}\times X_{3}+0.092500X_{1}\times X_{2}^{2} \end{array}$$

(5) $$\begin{array}{cc} Y_{2} & =8.03500+0.458250X_{1}+0.010125X_{2}+0.143750X_{3}\\ & ‐0.003500X_{1}\times X_{2}‐0.039250X_{1}\times X_{3}‐0.006750X_{2}\times X_{3}\\ & ‐0.277000X_{1}^{2}‐0.00500X_{2}^{2}‐0.181250X_{3}^{2}\\ & +0.109500X_{1}^{2}\times X_{3}‐0.048750X_{1}\times X_{2}^{2} \end{array}$$

where the (*–*) and (+) signs indicate antagonistic and synergistic effects, respectively. For fitness to fit a good model, a test for the significance of the regression model and individual model coefficients with lack‐of‐fit effect into the model is presented in Table 2. The data had been viewed in a way with a lack‐of‐fit that fits a model and adjusts for the effects of the center points and tests if there is a lack‐of‐fit in the center of the design space. Usually, the significant factors were ranked based on the *f*‐ or *p*‐value (probability value) with a 95% confidence level.

**TABLE 2:** ANOVA‐based data results for the response surface quadratic model for *Bifidobacterium breve* M4A and *Bifidobacterium longum* subsp. *longum* FA1 strains

Source	*Df*	Sum of squares		Mean square		*F*‐value		*p*‐Value		Statistical value
		*B. breve* M4A	*B. longum* subsp. *longum* FA1	*B. breve* M4A	*B. longum* subsp. *longum* FA1	*B. breve* M4A	*B. longum* subsp. *longum* FA1	*B. breve* M4A	*B. longum* subsp. *longum* FA1	
Model	11	4.83	2.35	0.4393	0.2133	308.55	344.34	<0.0001	<0.0001	Significant
*X*_1_	1	0.3522	0.8400	0.3522	0.8400	247.40	1356.05	<0.0001	<0.0001	
*X*_2_	1	0.0755	0.0008	0.0755	0.0008	53.00	1.32	0.0008	0.3019	
*X*_3_	1	0.0219	0.0827	0.0219	0.0827	15.38	133.44	0.0112	<0.0001	
*X*_1_ *X*_2_	1	0.1636	0.0000	0.1636	0.0000	114.92	0.0791	0.0001	0.7898	
*X*_1_ *X*_3_	1	0.3764	0.0062	0.3764	0.0062	264.35	9.95	<0.0001	0.0253	
*X*_2_ *X*_3_	1	0.2285	0.0002	0.2285	0.0002	160.48	0.2942	<0.0001	0.6108	
$X_{1}^{2}$	1	1.15	0.3231	1.15	0.3231	809.37	521.56	<0.0001	<0.0001	
$X_{2}^{2}$	1	0.0069	0.0001	0.0069	0.0001	4.83	0.2056	0.0794	0.6692	
$X_{3}^{2}$	1	0.5997	0.1383	0.5997	0.1383	421.21	223.31	<0.0001	<0.0001	
$X_{1}^{2}$ *X*_3_	1	0.4182	0.0240	0.4182	0.0240	293.69	38.71	<0.0001	0.0016	
*X*_1_ $X_{2}^{2}$	1	0.0171	0.0048	0.0171	0.0048	12.02	7.67	0.0179	0.0394	
Residual	5	0.0071	0.0031	0.0014	0.0006					
Lack of fit	1	0.0029	0.0011	0.0029	0.0011	2.79	2.15	0.1701	0.2169	Not significant
Pure error	4	0.0042	0.0020	0.0010	0.0005					
Cor total	16	4.84	2.35							
Std. dev.		0.0377	0.0249							
Mean		7.84	7.82							
C.V. %		0.4812	0.3184							
*R* ^2^		.9985	.9987							
Adjusted *R* ^2^		.9953	.9958							
Predicted *R* ^2^		.9943	.9741							
Adeq. precision		52.8671	68.0525							

The ANOVA test‐based analysis demonstrated that all three fortified JA juice (*X*_1_, *X*_2_, and *X*_3_) samples and their interaction could be correctly incorporated in the final model of both strains. *X*_2_ exhibited a minor influence on the *B. breve* M4A cell concentration as compared to control samples. Fortified JA samples *X*_1_, *X*_3_, and *X*_1_ *X*_2_ *X*_3_ had a notable impact on the *B. longum subsp. longum* FA1 cell concentration in contrast to the minor ones of *X*_2_, *X*_1_ *X*_2_, *X*_1_ *X*_3_, and *X*_2_ *X*_3_. Oxygen toxicity is considered an important factor responsible for bifidobacterial cell death. A previous study showed that the microaerophilic and anaerobic environment reduced the rate of persistence of probiotic bacteria, (*L. acidophilus* and *Bifidobacterium* spp.) that impacted on reducing the shelf life of yogurt (Talwalkar & Kailasapathy, 2004). Moreover, our study results revealed that the fortified JA juice protected the bifidobacteria against oxygen stress that may impact increasing the shelf life of fortified JA juice. The findings align with a previous study where response surface analysis showed that the optimum amount was 3.0%, 2.36%, and 4.99% for ascorbic acid, sodium erythorbate, and xylo‐oligosaccharide, respectively. The encapsulation yield of *B. bifidum* (BB01) reached to 94.88% from 90%, with a 21.6% increase in the viable counts. The results indicate that the model proves the adequacy of the regression that adequately fitted to the experimental data (Li et al., 2018).

### 3.5 Analysis of fortified JA juice for sensory assessment

The appearance of fortified JA juice was turbid with light brown color similar to the color of turbid apple juice showing a semi‐thick texture. The taste of fortified JA juice revealed sweet to slightly acidic savor and astringent with a characteristic aroma, which indicates the presence of different aromatic compounds in the JA tubers. The sensory scoring results of each type of fortified JA juice sample were measured by panelists as illustrated in Table 3. The results showed that ascorbic acid had the highest score followed by ginseng extract with organoleptic production that fermented with probiotic strains as compared to the astaxanthin (Table 3).

**TABLE 3:** Sensory data analysis of Jerusalem Artichoke juice fortified with ascorbic acid (*X*_1_), astaxanthin (*X*_2_), and ginseng extract (*X*_3_) after 4‐week storage at 5°C

Strain	Code of treated sample	Sensory criterion				
		Color	Texture	Flavor	Overall acceptance	Notes
*Bifidobacterium breve* M4A	Control	2.55 ± 0.52^a^	2.77 ± 0.65^a^	2.33 ± 0.50^a^	2.88 ± 0.60^a^	Satisfactory
	*X*_1_	2.66 ± 0.50^a^	2.88 ± 0.60^a^	3.44 ± 0.52^a^	3.33 ± 0.50^a^	Good
	*X*_2_	3.55 ± 0.53^b^	2.89 ± 0.60^a^	2.66 ± 0.50^ab^	2.78 ± 0.44^ab^	Satisfactory
	*X*_3_	3.11 ± 0.60^ab^	2.78 ± 0.44^a^	3.22 ± 0.65^b^	2.89 ± 0.33^ab^	Satisfactory
	*X*_1_ *X*_2_	3.00 ± 0.50^ab^	2.78 ± 0.44^a^	3.22 ± 0.44^b^	3.10 ± 0.33^ab^	Good
	*X*_1_ *X*_3_	3.00 ± 0.50^ab^	2.89 ± 0.33^a^	3.22 ± 0.44^b^	3.22 ± 0.44^ab^	Good
	*X*_2_ *X*_2_	3.55 ± 0.52^b^	2.77 ± 0.66^a^	3.00 ± 0.50^a^	3.11 ± 0.33^ab^	Good
	*X*_1_ *X*_2_ *X*_3_	3.22 ± 0.65^ab^	3.22 ± 0.44^a^	3.44 ± 0.52^b^	3.22 ± 0.44^b^	Good
*Bifidobacterium longum* subsp. *longum* FA1	Control	2.66 ± 0.50 ^a^	2.89 ± 0.78^a^	2.22 ± 0.44^a^	2.89 ± 0.60^a^	Satisfactory
	*X*_1_	2.77 ± 0.44^a^	3.00 ± 0.71^a^	3.44 ± 0.53^c^	3.33 ± 0.50^a^	Good
	*X*_2_	3.55 ± 0.52^b^	2.89 ± 0.33^a^	2.66 ± 0.50^ab^	2.77 ± 0.44^a^	Satisfactory
	*X*_3_	3.00 ± 0.50^ab^	2.88 ± 0.60^a^	3.22 ± 0.66^bc^	3.00 ± 0.50^a^	Good
	*X*_1_ *X*_2_	2.77 ± 0.44^a^	2.78 ± 0.44^a^	3.22 ± 0.44^bc^	3.00 ± 0.50^a^	Good
	*X*_1_ *X*_3_	3.11 ± 0.60^ab^	2.89 ± 0.33^a^	3.33 ± 0.50^bc^	3.11 ± 0.33^a^	Good
	*X*_2_ *X*_2_	3.44 ± 0.53^b^	3.00 ± 0.50^a^	3.00 ± 0.50^bc^	3.00 ± 0.50^ab^	Good
	*X*_1_ *X*_2_ *X*_3_	3.33 ± 0.50^ab^	3.22 ± 0.44^a^	3.55 ± 0.57^a^	3.33 ± 0.50^a^	Good

Note

^a,b,c^different letters indicate significant differences (*p* < .05). Sensory scale: 4.1–5.0 = very good; 3.1–4.0 = good; 2.1–3.0 = satisfactory; 1.1–2.0 = sufficient; 0.0–1.0 = not acceptable.

Overall, a combination of fortified JA juice *X*_1_ *X*_2_ *X*_3_ showed a significantly (*p* < .05) high acceptance as compared to the control JA juice simulated with *B. breve* M4Astrain. The highest mean color grade 3.55 (fair to good) was observed in the *X*_2_ and *X*_2_ *X*_3_ fortified JA juice as compared to the control (2.55). The texture score of the fortified JA juice samples had a similar rate of high acceptance. The flavor was graded 3.44 in the combination of *X*_1_ *X*_2_ *X*_3_ fortified JA juice sample, which presented the highest value as compared to the control sample. In contrast, there was no significant differences in the overall acceptance and texture scores for all fortified JA juice samples. The highest mean color was 3.55 observed in individual (*X*_2_) and combination (*X*_2_ *X*_3_) as compared to control (2.55) sample. The mean flavor (3.44) was significantly (*p* ˂ .05) different in *X*_1_ as compared to the control (2.22) sample.

The sensory evaluations showed no significant differences among various fortified JA juice samples. The improvement of sensory properties was enhanced by the addition of ascorbic acid, astaxanthin, and ginseng extract. The previous study has shown that the probiotics have minimal effect on the overall acceptance of juices (Perricone et al., 2015). An overall acceptability of fortified JA juices simulated with both bifidobacterial strains was observed with the benefit of the bifidobacterial cultures in JA juice that could add value in terms of health aspects (Alsharafani, 2006). Overall, the results indicate that there is suitable potential of supplements in the development of flavors for future use.

### 3.6 Development of regression model

**FIGURE 3:**
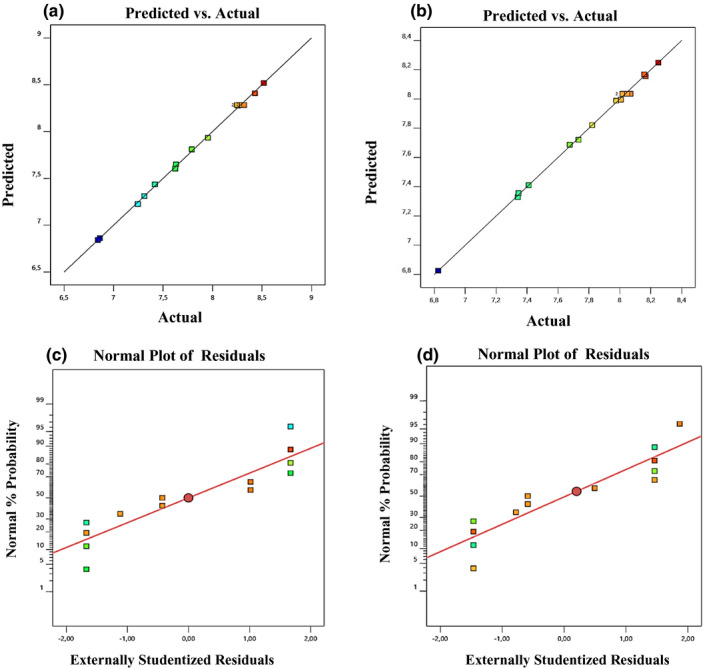
Diagnostic plot for developed model adequacy in actual response vs. predicted response for *Bifidobacterium breve* M4A (a) and *Bifidobacterium longum* subsp. *longum* FA1 (b) strains. The internally studentized residual vs. predicted strain numbers of the *B. breve* M4A (c) and *B. longum* subsp. *longum* FA1 (d)

The added factors (ascorbic acid, astaxanthin, and ginseng extract) were correctly described by the quadratic model of fortified JA juices on the response. The cell concentrations of both strains predicted by the ANOVA test formed a regression curve in actual versus predicted response (Figure 3a,b). Similarly, Figure 3c,d shows the internally studentized residuals versus predicted strain cell concentration. This signifies all values of response and adequacy of the developed model. In other terms, the experimental data sufficiently fitted to the model and can be used to test, evaluate, and predict the relationships among significant variables for obtaining the desired rate of persistence of bacterial cell concentration during 4 weeks of storage at 5°C. It has been reported that fruit juices have potential to be used as a suitable medium for the growth and persistence of bifidobacteria and *Lactobacillus plantarum* c19, where *B. animalis* subsp. *lactis* can significantly increase shelf life (26 days at 4°C) in red‐fruit juice, as compared to apple juice simulated with *L. plantarum* c19. On the other hand, citrus extract (e.g., biocitro) can be used as a natural preservative in fruit juices since probiotics showed resistance to biocitro; moreover, biocitro may also improve the biocontrolling action of *L*. *plantarum* against *Zygosaccharomyces bailii*. However, probiotics showed no effect on sensory properties (Bevilacqua et al., 2013).

**FIGURE 4:**
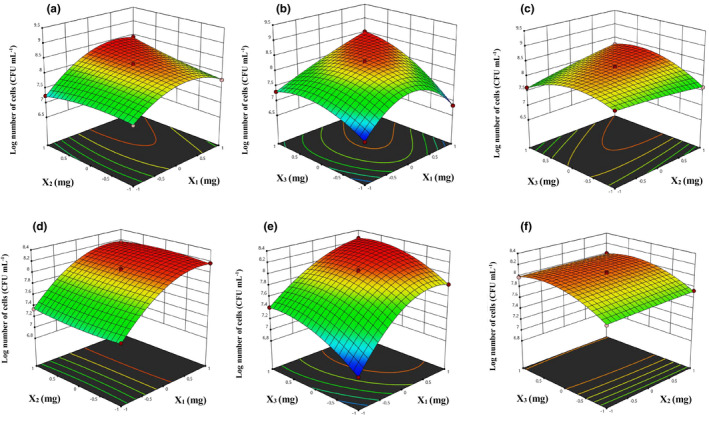
Response surface design of Log number of cells (CFU/ml) for *Bifidobacterium breve* M4A (models a–c) and *Bifidobacterium longum* subsp. *longum* FA1 (models e–f). Where *X*_1_ *X*_2_ represented in models a & b; *X*_1_ *X*_3_ illustrated in models b & e and *X*_2_ *X*_3_ illustrated in models c & f. However, the third variable of each graph was kept constant at the central point

**FIGURE 5:**
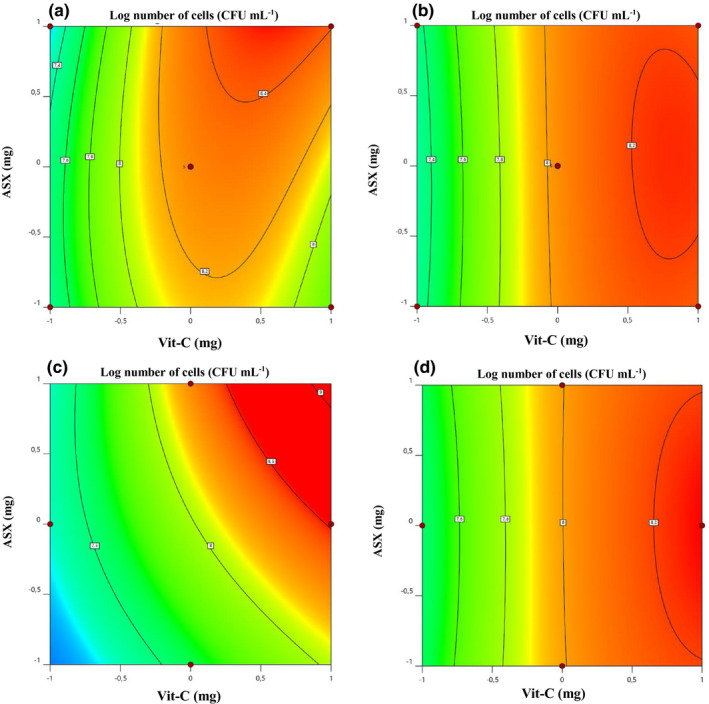
Models a & b represent two‐dimensional contour plot of factors *X*_1_ vs. *X*_2_ without adding prediction power effect of *X*_3_, a mid‐level slice (0 level) of the coded level third factor of *Bifidobacterium breve* M4A and *Bifidobacterium longum* subsp. *longum* FA1 strains. Models c & d represent the graph indicating currently shown surface hot of the third‐factor effect of *X*_3_ at a higher coded level (+1) on the contour plot of *B. breve* M4A and *B. longum* subsp. *longum* FA1 strains

As shown in Table 2 where *X*_1_ sample had a greater effect on *B. breve* M4A and *B. longum* subsps. *longum* FA1 cell concentration with a high *f‐*value (5849.88 and 680.05) than other variables. Similarly, the effect of the combination of *X*_1_ *X*_2_, *X*_1_ *X*_3_, and *X*_2_ *X*_3_ fortified JA juices on bacterial cell concentration was significantly high as compared to the control JA juice spiked with *B. breve* M4A strain (Figure 4; models a–c). However, the bacterial cell concentration was not significantly affected by combinations *X*_1_ *X*_2_ and *X*_2_ *X*_3_ of fortified JA samples spiked with *B. longum* subsps. *longum* FA1 strain (Figure 4; models d–f). Two‐dimensional contour plots demonstrated the interaction of components in the combination of *X*_1_ *X*_2_ *X*_3_ fortified JA juice which has the most effective combination for gaining high bacterial cell concentration in JA juice (Figure 5).

### 3.7 Assessment of regression model

For assessing the reliability of the regression model in terms of lack‐of‐fit for *B. breve* M4A and *B. longum* subsp. *longum* FA1, the precision of model was evaluated by coefficient of determination *R* ^2^ (Table 2). The *R* ^2^ values (.9985 and .9987) for *B. breve* M4A and *B. longum* subsp. *longum* FA1, respectively, confirmed that only 0.9% of the entire response variation was not described by the model. The adjusted *R* ^2^ values (.9953 and .9958) were in a reasonable agreement with *R²* values indicate that the model had related variables for *B. breve* M4A and *B. longum* subsp. *longum* FA1. The reproducibility and fitness of model were also assigned in terms of coefficient of variation (CV). The CV (0.4812% and 0.3184%) confirmed the efficient reproducibility of the model for *B. breve* M4A and *B. longum* subsp. *longum* FA1, respectively. The quadratic model exhibits low standard deviation, high *R* ^2^ values, which is acceptable for the suggested quadratic model. The factorial design with center points showed a significant curvature by the points that are sticking above the surface plot (Figure 4). Design Expert v.12 provides special treatment for center points, which show the lack‐of‐fit is not significant. Therefore, Design‐Expert by a special handling of center points provides a much more enlightening view of the design (Anderson et al., 2015).

According to the ANOVA test analysis, the results of the obtained model were facilitated to study the effects of fortified factors of the three‐dimensional response surfaces plot. A positive association of *X*_1_ and *X*_2_ with *B. breve* M4A cell concentration has been observed (Figure 4a). However, a significantly high impact of *X*_1_ and *X*_3_ on the cell concentration can be explained as higher both strains' cell concentrations can be obtained in comparison to the control. In addition, the effect of adding *X*_2_ and *X*_3_ on the bacterial cell concentration is illustrated in Figure 4c. It can be summarized that both *X*_2_ and *X*_3_ are synergized and positively related to the growth of *B. breve* M4A and *B. longum* subsp. *longum* FA1 strains.

Similarly, a synergistic relationship between ascorbate and inulin on cell viability for eliminating the stress of remaining oxygen in treated juices that contained ascorbic acid was observed (Shah et al., 2010). The addition of ascorbic acid and l‐cysteine‐HCl showed a significant impact on the growth of *B. bifidum* BB01 and *B. bifidum* BB03 strains. Adding 0.8 g/L of ascorbic acid or 0.6 g/L of l‐cysteine‐HCl has a significant impact on the growth of bifidobacterial strains (Shu et al., 2013). The cellular uptake of astaxanthin nanodispersions in skimmed milk was significantly higher than astaxanthin nanodispersions in orange juice and deionized water. High in vitro cellular uptake of astaxanthin from the prepared astaxanthin nanodispersions can be achieved by the incorporation of protein‐based foods such as milk (Anarjan & Tan, 2013; Mezquita et al., 2014).

### 3.8 Interaction effect of variables on cell concentration and viability

The findings provided evidence that when the *X*_1_ is 15 g/L, in the mixture supplement, the CFU/ml of *Bifidobacterium* sp. was one log higher than strain in the *X*_2_ and the control JA juice samples. The interaction effect of *X*_1_ and *X*_3_ with the *Bifidobacterium* sp. cell concentration (CFU/ml) is presented in Figure 4 (models a and b). The total number of cells (CFU/ml) of both bifidobacterial strains remained unchanged in *X*_2_ JA juice samples, in comparison with the control sample, which may be explained by a limited bioavailability of *X*_2_ during the storage period.

The combination of *X*_1_, *X*_2_, and *X*_3_ fortified JA juice samples showed no effect on the cell concentration (CFU/ml) of *B. longum* subsp. *longum* FA1 (Figure 4; models d–f). The interaction effect of the combination *X*_1_ *X*_2_ *X*_3_ on the bifidobacterial cell concentration (CFU/ml) is illustrated in Table 1. The significant impact of *X*_1_, *X*_2_, and *X*_3_ on the bacterial concentration (CFU/ml) where a higher bacterial cells concentration than the control sample was observed. On the other hand, the cell concentration of *B. longum* subsp. *longum* FA1was significantly (*p* ˂ .05) higher in the combination of *X*_1_, *X*_3_, *X*_1_ *X*_3_, and *X*_1_ *X*_2_ *X*_3_ fortified JA juices than *X*_1_ *X*_2_ and *X*_2_ *X*_3_ after 28 days at 5ºC (Table 2).

The results show that the *Bifidobacterium* strains can maintain the desired cell concentration (ranging from 10^7^ to10^8^ CFU/ml) in the fortified JA juices, which indicate that JA juice itself is a suitable medium for the propagation of probiotics bacteria. Previous studies evaluated the viability of *L. paracasei*, *L. casei*, and *L. rhamnosus* in mango beverages stored at 4°C and *L. casei* was identified as the most stable strain when tested with a 5% fructooligosaccharides substance for growth stimulation and produced significant changes in pH and titratable acidity of the mango beverage after 1 week of storage at 4°C (Acevedo‐Martínez et al., 2018). Fructooligosaccharides of JA tuber has the potential to be used as a probiotic component because it can provide great stability to probiotics and acid production (Elaheh et al., 2016). However, probiotic viability should be at least 10^7^ CFU/ml in the product at the end of the shelf life (Nualkaekul et al., 2011). Previous study used microwave freeze‐dried at 25°C and microwave vacuum storage methods to enhance persistence of *B. animalis* subsp. *lactis* INL1 (Zacarias et al., 2020). The results suggest that dehydration method can also be applied in the food industry.

The survivability of probiotic bacteria in juices depends on various factors such as storage temperature during refrigeration that can prolong survival rate. For combating harmful effect of high temperature, various strategies including microencapsulation and reduce acidification methods have been investigated (Nag & Das, 2013; Sohail et al., 2012). Also, antioxidant molecules could also be a useful strategy for limiting the harmful effects of oxygen exposure by using plant‐based extracts (Nag & Das, 2013).

### 3.9 Optimization of JA juice with three factors

The two‐dimensional contour plot of factors *X*_1_ versus *X*_2_ including six center points is indicated by the dot in the middle of the contour plot of the *B. breve* M4A and *B. longum* subsp. *longum* FA1 strains (Figure 5a,b). As a result of two‐dimensional overviews of the contour plot, a contour plot of conversion as a function of *X*_1_ and *X*_2_ at a mid‐level slice of the coded level of *X*_3_, where *X*_3_ did not currently assign to axes or plotted on the graphs, whereas the third‐factor effect of *X*_3_ at a higher coded level (+1) on the contour plot of the *B. breve* M4A and *B. longum* subsp. *longum* FA1 strains at higher response levels (Figure 5c,d). This result is consistent with the regression analysis results, where *X*_1_, *X*_2_, and *X*_3_ had the highest regression coefficient.

The most interaction limitation in the combination of *X*_1_ *X*_2_ and *X*_2_ *X*_3_ fortified JA juice was observed against *B. longum* subsp. *longum* FA1 strain. Our results in the extension of storage are in agreement with the data reported by Dimitrovski et al. (2016), referring to the pure juice of JA that showed an excellent source of nutrients for the growth of *L. plantarum* PCS26 where the cell concentration reached to ˃10^10^ CFU/ml in 12 h. Culture survivability in the fermented juices mixed with 30% blueberry juice during storage between 4 and 7°C where blueberry enhanced the overall acceptance of the juice. The product shelf life was extended from 19 days of pure JA juice to 35 days supplemented with 30% blueberry juice. The potential for bifidobacteria fermenting fruit and vegetable juice was investigated, where *B. longum* Bb‐46 strain was grown well in orange, carrot, and tomato juices (Buruleanu et al., 2009). Nualkaekul et al. (2011) investigated different factors of acidic juices that control *B. longum* persistence in the model solutions and fruit juices. The bifidobacteria cells decreased (˂0.8 log_10_ CFU/ml) in orange, grapefruit, blackcurrant, and pineapple juices after 6 weeks of storage at 4°C. The highest cell concentration was observed in orange and pineapple juice.

## 4 CONCLUSION

The study has provided data on the persistence of both *Bifidobacterium* species that remained ˃10^7^ (CFU/ml) up to 28 days at 5ºC and produced a functional drink as compared to nonfortified JA juice. JA juice fortified with ascorbic acid (*X*_1_) showed a significantly high rate of persistence of probiotic bacteria (CFU/ml). The benefits (taste and shelf life) of *B. breve* M4A and *B. longum* subsps. *longum* FA1 had resulted in increasing their incorporation into JA juice supplemented with *X*_1_, *X*_2_, and *X*_3_ extracts to produce functional drinks. In the case of the examined JA juice supplemented with *X*_1_ and *X*_3_ served as appropriate supplements, whereas *X*_2_ was strain dependent. The findings of the surface response analysis were obtained on a laboratory scale and can eventually be used to various production volumes. The results would help food industry in producing JA juice fortified with antioxidant compounds that can be an alternative to dairy‐based probiotics and provide essential human nutrition. Studies are underway to further determine the effects of JA beverage fortified with ascorbic acid, astaxanthin, and ginseng extract on human health.

## CONFLICT OF INTEREST

The authors declare that the research was conducted in the absence of any commercial or financial relationships that could be construed as a potential conflict of interest.

## AUTHOR CONTRIBUTIONS

**Mustafa Alsharafani:** Formal analysis (lead); Funding acquisition (equal); Project administration (lead); Resources (equal); Supervision (lead). **Taghreed Abdullah:** Data curation (equal); Formal analysis (equal); Investigation (equal); Methodology (equal). **Zahraa A. Jabur:** Data curation (equal); Formal analysis (equal); Investigation (equal); Methodology (equal); Writing – review & editing (supporting). **Abdulwahed Ahmed Hassan:** Methodology (equal); Writing – review & editing (equal). **Abeer S. Alhendi:** Methodology (equal); Resources (equal); Writing – review & editing (supporting). **Amir Abdulmawjood:** Resources (equal); Writing – review & editing (supporting).

## Data Availability

The study data can be shared upon request from the corresponding author. The data are not publicly available due to privacy restrictions.
